# The validity of the patient health Questionnaire-9 to screen for depression in patients with type-2 diabetes mellitus in non-communicable diseases clinics in Malawi

**DOI:** 10.1186/s12888-019-2062-2

**Published:** 2019-02-27

**Authors:** Michael Udedi, Adamson S. Muula, Robert C. Stewart, Brian W. Pence

**Affiliations:** 1 Department of Mental Health, University of Malawi, College of Medicine, P/Bag 360, Chichiri, Blantyre, 3 Malawi; 2 Department of Clinical Services, Ministry of Health, P. O. Box 30377, Capital City, Lilongwe, 3 Malawi; 3 Department of Public Health, University of Malawi, College of Medicine, P/Bag 360, Chichiri, Blantyre, 3 Malawi; 4 Africa Center of Excellence in Public Health and Herbal Medicine, University of Malawi, College of Medicine, P/Bag 360, Chichiri, Blantyre, 3 Malawi; 5 Epidemiology Department, University of North Carolina at Chapel Hill Gillings School of Global Public Health, 135 Dauer Dr, Chapel Hill, NC 27599 USA

**Keywords:** PHQ-9, Validation, Non communicable diseases, Depression, Diabetes mellitus, Malawi

## Abstract

**Background:**

Depression is a global problem, affecting populations worldwide, but is too often under-diagnosed. The identification of depression among patients with diabetes is important because depression is prevalent in this group and can complicate diabetes management.

**Objectives:**

The aim of the study was to determine the sensitivity and specificity of the PHQ-9 in the detection of depression among patients with type-2 diabetes mellitus attending non-communicable diseases (NCD) clinics in Malawi.

**Methods:**

We conducted a validation study of the Patient Health Questionnaire (PHQ-9) among 323 patients with type-2 diabetes mellitus who attended two NCD clinics in one of the 28 districts of Malawi. The participants were screened consecutively using the nine-item PHQ-9 in Chichewa by a research assistant and completed a diagnostic interview using the Structured Clinical Interview for DSM-IV (SCID) for depression with a mental health clinician. We evaluated both content validity based on expert judgement and criterion validity of the Patient Health Questionnaire (PHQ-9) based on performance against the SCID. The PHQ-9 cutpoint that maximized sensitivity plus specificity was selected to report test characteristics.

**Results:**

Using the SCID for depression, the prevalence of minor or major depression was 41% (133/323). The internal consistency estimate for the PHQ-9 was 0.83, with an area under the receiver operator curve (AUC) of 0.93 (95% CI, [0.91–0.96]). Using the optimal cut-point of ≥9, the PHQ-9 had a sensitivity of 64% and a specificity of 94% in detecting both minor and major depression, with likelihood ratio-positive = 10.1 and likelihood ratio negative =0.4 as well as overall correct classification (OCC) rate of 81%.

**Conclusions:**

This is the first validation study of the PHQ-9 in NCD clinics in Malawi. Depression was highly prevalent in this sample. The PHQ-9 demonstrated reasonable accuracy in identifying cases of depression and is a useful screening tool in this setting. Health care workers in NCD clinics can use the PHQ-9 to identify depression among their patients with those having a positive screen followed up by additional diagnostic assessment to confirm diagnosis.

**Trial registration:**

PACTR201807135104799. Retrospectively registered on 12 July 2018.

**Electronic supplementary material:**

The online version of this article (10.1186/s12888-019-2062-2) contains supplementary material, which is available to authorized users.

## Background

Depression contributes significantly to the global burden of disease. Depression is often under-diagnosed in low and middle-income countries (LMICs) [1, 2]. The poor detection of depression is associated with disability and leads to increased use of health services for physical health complaints in both high-income countries and LMICs [3]. If healthcare workers are able to diagnose depression, they can reduce morbidity and improve patient wellbeing by providing cost-effective treatments [4, 5]. In order to detect and diagnose depression, we need to have effective tools. Because depression varies based on cultural context, screening tools must be adapted and validated for particular populations.

There are a number of tools used to screen for depression. The Patient Health Questionnaire (PHQ-9) is widely used for screening and monitoring treatment of depression [6]. The PHQ 9 is a nine-item scale assessing symptoms experienced in the preceding two weeks. The reliability and validity of the PHQ-9 are sound, and internal validity of the PHQ-9 is high. The PHQ-9 questions are easily understood, and the PHQ-9 requires minimal time to administer and score [7]. The PHQ-9 has been validated and translated in some African countries including Nigeria [8], Ghana [9], Kenya [10], Cameroon, [11], Ethiopia [12], South Africa [13], and Uganda [1]. However, the PHQ-9 has not been validated for use in Malawi, and there are currently few tools for efficient and effective depression screening in a general healthcare setting in Malawi. The Self Reporting Questionnaire (SRQ) has been validated to screen for major depressive episodes in Malawi [14]. However the SRQ-20 is limited in response options (‘yes’ or ‘no’) [15], whereas the PHQ-9 has greater variety of options for describing symptom occurrence.

Accordingly, we conducted a validation study of the PHQ-9 for detection of depression in Malawi. We validated this tool in patients with type-2 diabetes mellitus attending two NCD clinics in Malawi. We chose the PHQ-9 for this study because it is effective in other settings and brief, which is compatible with Malawian health care setting workload. We conducted this study in an NCD clinic because depression is often comorbid with NCDs [16–18], and it is particularly important to have a tool that is validated for use in general healthcare settings, such as an NCD clinic. We included only patients with diabetes because the health care burden associated with the rapidly increasing diabetes population in Malawi, and the concern that depression can interfere with clinic appointment attendance and treatment adherence, makes this a timely and important focus [19–23]. The use of a valid screening tool for depression will help clinicians better diagnose patients and initiate treatment, which is in line with the strategy of integration of mental health as outlined in Malawi’s National Mental Health Policy.

## Materials and methods

### Setting and participants

We conducted the study in Lilongwe district, a predominantly Chichewa speaking district. The study was conducted in two NCD clinics of Area 25 Health Centre under Lilongwe District Health Office and Kamuzu Central Hospital. The Area 25 Health Centre has been piloting a chronic care clinic for key NCDs such as hypertension, diabetes, asthma and epilepsy at the primary health care level since March 2014. Research assistants recruited consecutive patients attending the NCD clinic at the study sites between December 2017 and April 2018. Participants were eligible for the study if they were at least 18 years of age or older, attending the NCD clinic for diabetes care, and available for an interview. Participants were excluded if they required acute medical care or were unable to speak.

### Validation

#### Content validity

The PHQ-9 is a depression module that incorporates the Diagnostic and Statistical Manual of Mental Disorders (DSM) criteria into a brief measure of depression [24]. The PHQ-9 is a concise tool for assessing depression. Two bilingual Malawians translated the English PHQ-9 independently into Chichewa; one mental health nurse and one linguistics and communication specialist. This was followed by evaluation of the translated tools by the principal investigator, two mental health professionals and two health promotion officers with extensive expertise in developing health communication tools in order to arrive at a consensus translation. Two additional independent bilingual Malawians back-translated the consensus Chichewa translation into English.

We pretested the Chichewa translated version of the PHQ 9 on a convenience small sample of 15 participants attending a general outpatient clinic in area 25. The PHQ-9 was interviewer-administered, and participants were probed about their perceived interpretation of the constructs. This pretesting helped to identify any challenges respondents might have with the translation. Any unclear Chichewa terms were modified to include terms that are more commonly used and understandable by the participants in order to produce a final PHQ-9 Chichewa translation. (Additional file 1).

#### Criterion validity

The Structured Clinical Interview for DSM-IV (SCID) [25] depression module was used as a gold standard to validate the PHQ 9. The SCID is a semi-structured interview designed for administration by a clinician or skilled researcher that determines formal diagnosis according to the Diagnostic and Statistical Manual of Mental Disorders. The SCID for depression was translated into Chichewa previously and has been used in Malawi after undergoing a process of validation which included translation, back translation and testing [14] . At the time of the study the most recent version of SCID was not available however major depression is defined in both the DSM-IV and DSM-V as the presence of either depressed mood or loss of interest or irritability with five or more depressive symptoms, lasting at least two weeks, with no history of a manic, hypomanic, or mixed episode [26, 27]. In contrast, minor depression is described in the DSM-IV as the presence of at least two, but less than five, depressive symptoms (one symptom must be either depressed mood or loss of interest) during the same 2-week period, with no history of a major depressive episode or dysthymia [27].

### Study procedure

The sample size was calculated using Buderer’s formula [28]. We used the following parameters: anticipated sensitivity (*SN*) of the PHQ-9 was 80%, the standard normal deviation corresponding to the specified size of the critical region (z^2^_1_-α/2) is 3.84, alpha (*α)* which is size of the critical region is 0.05, the absolute precision desired on either side of sensitivity L was set at 0.1, and prevalence for depression was estimated as 20%. The prevalence of depression of 20% was based on a recent study among patients attending a health care setting in Malawi [29] . We increased the sample size by 5% to account for potential participants’ refusal and loss of data. Taking these assumptions into consideration, the required sample size was calculated as 323.

Two research assistants conducted the recruitment and screening of participants. The research assistants had bachelor’s degree and had 3 days of training in administration of the research tools and spoke both English and Chichewa. After assessing each patient for inclusion and exclusion criteria, the research assistants explained the study’s purpose and procedures. Informed consent (signed or thumb printed) was obtained from patients. The research assistant collected demographic information from the participant and then administered the Chichewa version of the PHQ-9 in a private room.

After seeing their clinician for their regular appointment, participants completed the SCID with a separate SCID interviewer. The SCID interviewer was masked from the PHQ-9 score. The SCID interviewer was a mental health clinician and had regular supervision and record review for quality assurance to ensure consistence and accuracy of diagnoses. Finally, the patient’s health passport was examined to determine whether or not the NCD clinician had made a diagnosis of depression and/or prescribed antidepressant medication during the clinical encounter.

### Data analyses

We compared the PHQ-9 score against the “SCID” diagnosis of depression that was made by the 2-stage diagnostic process. We also calculated the sensitivity, specificity, positive predictive value (PPV), and negative predictive value (NPV) for various PHQ-9 cut-off scores. The PHQ-9 ability to discriminate between cases and non-cases was then examined using receiver operating characteristic (ROC) analysis. The ROC curve analysis was used to choose cut-points for the PHQ-9 scale. Two different cut-points were used, and the diagnostic ability was assessed by a number of statistics at each of these points. The first cut-point was the point which maximised the combination of sensitivity and specificity, whilst the second cut-point was chosen to give a more give a higher sensitivity. The PHQ-9 cutpoint that maximized sensitivity + specificity was selected to report test characteristics. The ROC curves were obtained by plotting sensitivity against 1-specificity for each possible cut-off score. The area under the ROC curve (AUC) was used to indicate the performance of the PHQ-9. A value of 0.5 on the AUC indicates discrimination no better than chance, and a value of 1.0 represents perfect discrimination. The correctly classified rate and likelihood ratio were also considered. Internal consistency of the PHQ-9 was evaluated using Cronbach’s alpha. Data analysis was conducted using SPSS version 20.0.

### Ethical approval

We obtained ethical approval from the University of Malawi, College of Medicine Review and Ethics Committee (COMREC) (Reference-P.07/17/2218). Written informed consent was obtained from every participant and fingerprint impressions were taken from consenting illiterate participants. All interviews were conducted in private at the health facilities.

## Results

### Sample characteristics

In total, 323 patients who were eligible and approached completed both the PHQ-9 and SCID between December, 2017 and April, 2018 thus we had no refusals. Among the 323 patients, 127 (39.3%) had diabetes only while 196 (60.7%) had both diabetes and hypertension. The mean patient age was 54 years (range, 21–79 years), with a standard deviation of 11.4 years; 75.5% of patients were female (Table 1).

**Table 1:** Socio-demographic characteristics of 323 validation study participants recruited from two non-communicable diseases clinics in Lilongwe, Malawi

Age	Mean (SD)
Mean age in years	53.8 (11.4)
Gender	n (%)
Female	244 (75.5)
Male	79 (24.5)
Education	
No education	27 (8.4)
Primary school	153 (47.4)
Secondary	127 (39.3)
Tertiary	16 (5.0)
Marital status	
Married	249 (77.0)
Never married	8 (2.5)
Widowed	50 (15.5)
Separated/divorced	16 (5.0)
Employment Status	
Unemployed	179 (55.4)
Student	1 (0.3)
Employed	64 (19.8)
Micro & Small scale Enterprise	79 (24.5)
Religious affiliation	
Christian	305 (94.4)
Moslem	17 (5.3)
None	1 (0.3)

### Prevalence of depression

Of the 323 patients, a total of 133 had either a minor or major depression identified by SCID, resulting in an estimated prevalence of 41%. Major depression was observed in 58 (18%) of the patients. No patients (0%) were diagnosed with depression or prescribed depression treatment by the NCD clinicians.

### Performance of the patient health Questionnaire-9

**Fig. 1:**
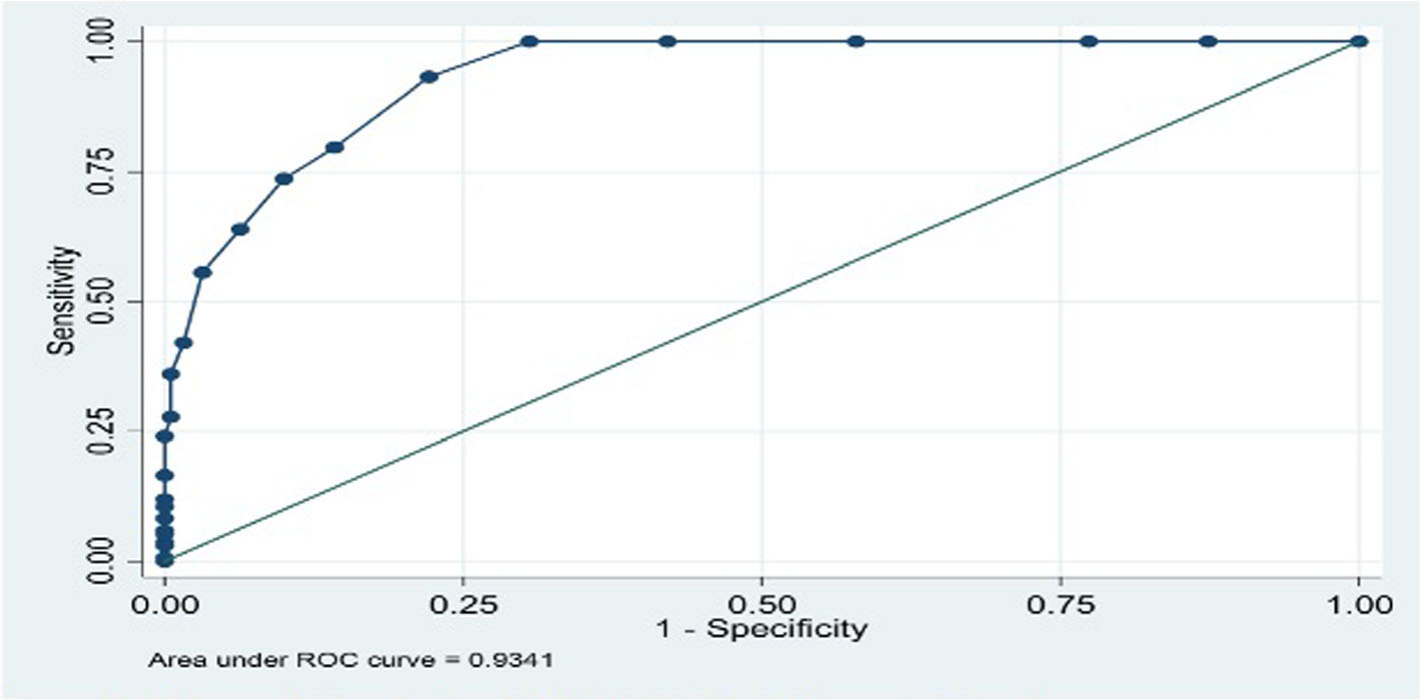
The predictive ability of PHQ-9 for detecting any depression

The calculated internal consistency of the PHQ-9 (Cronbach’s alpha) was 0.83. The area under the receiver operator curve was 0.93 (95% CI, [0.91–0.96]) (Fig. 1), suggesting good discriminating power of the PHQ-9 between cases and non cases of depression.

The results suggested that a cut-point of 6 or higher on the PHQ-9 scale gave the best combination of sensitivity and specificity in detecting either minor or major depression. This cut-point gave a very high sensitivity of 93% and a lower specificity of 78%. There was a high NPV of 94%, but a lower PPV of 74%. The Overall Correct Classification (OCC) rate was 84% at a cut point of 6 or higher and the likelihood ratio of a positive screen for depression was 4.2) and the likelihood ratio of a negative screen was 0.9. Using a higher cut-off of ≥7 increased the specificity to 86%. However, this was at the expense of sensitivity, which dropped down to 80%. There was a high NPV of 86%, but a lower PPV of 79% with an OCC of 83% and likelihood ratio-positive of 5.6.

The best combination of sensitivity and specificity was found to be at a cut-point of 9 or higher. A cut-point of ≥9 had a sensitivity of 64% and a specificity of 94% in detecting either minor or major depression. At the cut-point of ≥9, the NPV was 79% while the PPV was 88% and the likelihood ratio-positive was 10.1 and the likelihood ratio-negative was 0.4. The OCC rate of 81% was also good at a cut point of 9 and higher (Table 2).

**Table 2:** Operating characteristics of the Patient Health Questionnaire-9 at various cut-off scores for identifying either minor or major depression

Cut point	Sensitivity	Specificity	Correctly Classified	LR+	LR -
(> = 4)	100.00%	57.89%	75.23%	2.3750	0.0000
(*>* = 5)	100.00%	69.47%	82.04%	3.2759	0.0000
(*>* = 6)	93.23%	77.89%	84.21%	4.2177	0.0869
(*>* = 7)	79.70%	85.79%	83.28%	5.6085	0.2366
(*>* = 8)	73.68%	90.00%	83.28%	7.3684	0.2924
(*>* = 9)	63.91%	93.68%	81.42%	10.1190	0.3852
(*>* = 10)	55.64%	96.84%	79.88%	17.6190	0.4581
(*>* = 11)	42.11%	98.42%	75.23%	26.6668	0.5882
(*>* = 12)	36.09%	99.47%	73.37%	68.5717	0.6425
(*>* = 13)	27.82%	99.47%	69.97%	52.8573	0.7256

LR+ = Likelihood Ratio Positive LR- = Likelihood Ratio Negative

**Fig. 2:**
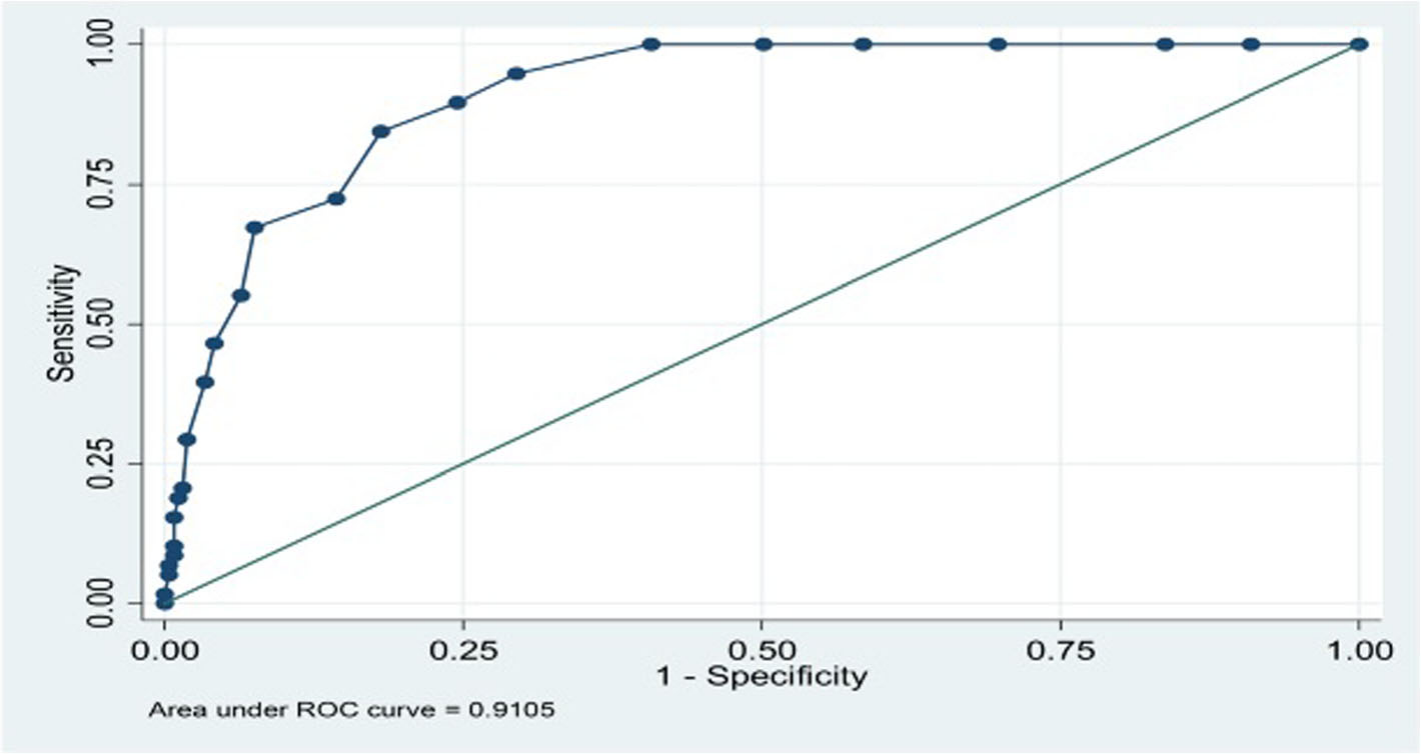
The predictive ability of PHQ-9 for detecting Major Depression

A similar analysis was performed to examine the predictive ability of PHQ-9 for detecting major depression alone. The ROC curve analysis gave an AUC value of 0.91 (95% CI, 0.88 to 0.94; Fig. 2).

The best combination of sensitivity and specificity was found to be at a cut-point of 9 or higher. This gave a relative high sensitivity and specificity of 85 and 82% respectively. There was also very high NPV with a value of 96%, but a lower PPV of 51% with an OCC of 82% and likelihood ratio-positive of 4.6. Lowering the cut-point on the PHQ-9 scale to 7 gave relatively similar overall performance in terms of the combination of sensitivity and specificity. However, using this cut-off increased sensitivity up to 95%, but at the expense of specificity which dropped to 71%. The OCC was 75% and likelihood ratio-positive was 3.2 (Table 3).

**Table 3:** Operating characteristics of the Patient Health Questionnaire-9 at various cut-off scores for identifying major depression

Cut point	Sensitivity	Specificity	Correctly Classified	LR+	LR -
(> = 4)	100.00%	41.51%	52.01%	1.7097	0.0000
(*>* = 5)	100.00%	49.81%	58.82%	1.9925	0.0000
(*>* = 6)	100.00%	59.25%	66.56%	2.4537	0.0000
(*>* = 7)	94.83%	70.57%	74.92%	3.2217	0.0733
(*>* = 8)	89.66%	75.47%	78.02%	3.6552	0.1371
(*>* = 9)	84.48%	81.89%	82.35%	4.6642	0.1895
(*>* = 10)	72.41%	85.66%	83.28%	5.0499	0.3220
(*>* = 11)	67.24%	92.45%	87.93%	8.9095	0.3543
(*>* = 12)	55.17%	93.58%	86.69%	8.6004	0.4790
(*>* = 13)	46.55%	95.85%	87.00%	11.2147	0.5576

LR+ = Likelihood Ratio Positive LR- = Likelihood Ratio Negative

## Discussion

Few screening tool to detect common mental disorders (CMDs) have been specifically developed in low and middle income countries [30] as such many researchers rely on tools from developed countries. It is important that instruments for screening patients have to be evaluated for their reliability and validity prior to their use in a country to ensure that the instruments are measuring what they supposed to measure [31].

In this study when the PHQ-9 was used against the gold-standard diagnosis, it performed well showing reasonable accuracy in identifying cases of depression. The area under the ROC curve was found to be 0.93, this is a high value, suggesting good diagnostic ability of the PHQ-9 score.

In this study, the PHQ-9 showed good predictive performance, comparable to that seen in validation studies in other parts of sub-Saharan Africa [1, 13]. The internal consistency observed for the PHQ-9 was also similar to that found in other previous study elsewhere [32]. Maximum sensitivity with a specificity ≥75% has been considered as desirable for clinical use [32]. Relative to previous studies, our results suggest PHQ-9 has better sensitivity and acceptable specificity in the type 2 diabetic population.

Depression is common and likely to impact diabetes mellitus care therefore routine screening is really important in diabetes mellitus care and we need these tools. In this validation study of the PHQ-9 in NCD clinic settings in Malawi, there was evidence of high prevalence of depression in patients with diabetes mellitus. The rate of depression in this study is comparable to rates of depression in other LMICs such as 46% in South Africa, 40% in Iraq, 32% in Egypt, 15 to 30% in Nigeria, 14. 7 to 43% in Pakistan, 43 to 70% in Iran, 27 to 63% in Mexico [33] and 39.73% in Ethiopia [34]. Depression often is missed by clinicians working in NCD clinics. Indeed, none of the cases of SCID-defined minor or major depression had been identified by the patient’s NCD clinician. This underscores the importance of this study. Given that the study has shown that identifying depression in NCD clinic is a challenge, a short and valid screening tool like PHQ-9 can assist in the identification of patients with depression.

One of the strength of this study is that it is the first study to consider the validity of the PHQ-9 in non communicable disease clinics in Malawi, and the first to validate the PHQ-9 in Malawi. Another strength of the study, is the careful attention which was paid to the translation. The study also used a reference standard, the SCID for depression that had previously been translated and adapted for use in Chichewa. A limitation of this study is that the participants were drawn from only two specialized NCD clinics in Lilongwe which may not be representative of the wider population.

## Conclusion

The validity, ease of administration and brevity of the PHQ-9 imply that it will be a valuable tool for identifying comorbid depression in patients with non communicable diseases using a cut-point of ≥9. Clinicians in NCD clinics when choosing a tool for screening depression should consider the PHQ-9. The use of a validated PHQ-9 will be in line with the strategy of integrating depression management in chronic care clinics in Malawi. The findings support our planned use of the PHQ-9 as a screening tool in a pilot study to evaluate the effects of depression management on glycaemic control in non communicable diseases clinics in Malawi.

## Additional file

Additional file 1: The Chichewa Patient Health Questionnaire (PHQ-9). (DOCX 18 kb)

## Abbreviations

COMREC: College of Medicine Research and Ethics Committee
DSM: Diagnostic and Statistical Manual of Mental Disorders
NCDs: Non-communicable Diseases
NPV: Negative Predictive Value
OCC: Overall Correct Classification
PHQ-9: Patient Health Questionnaire-9
PPV: Positive Predictive Value
SCID: Structured Clinical Interview for DSM
