# Supplementary material for: The validity of the patient health Questionnaire-9 to screen for depression in patients with type-2 diabetes mellitus in non-communicable diseases clinics in Malawi
Source: BMC Psychiatry. 2019 Feb 27;19:81. doi: 10.1186/s12888-019-2062-2 (PMC6391834; doi:10.1186/s12888-019-2062-2)
Supplement: Supplementary file 1 — The Chichewa Patient Health Questionnaire (PHQ-9). (DOCX 18 kb) [file 12888_2019_2062_MOESM1_ESM.docx]

Additional file 1: The Chichewa Patient Health Questionnaire (PHQ-9).

| M’masabata awiri apitawa ndi kangati mwakhala ndi chizindikiro chirichonse mwa zizindikiro izi? | Sindinavutikepo/  sindinakhalepo  Palibe ndi tsiku limodzi lomwe | Kus aposera sabata imodzi)  Masiku  1-7 | Kuposera sabata imodzi  Masiku  8-12 | Pafupifupi tsiku lirilonse  Masiku 13 kapena 14 |
| --- | --- | --- | --- | --- |
| 1. ***M’masabata awiri apitawa***, ndikangati mwakhala okhumudwa kapena kukhala opanda chiyembekezo (kutaya mtima) kapena nkhawa? | 0 | 1 | 2 | 3 |
| 2. ***M’masabata awiri apitawa***, ndikangati munavutika kukhala ndi chidwi chochepa kapena chilakolako pa zinthu (kusowa mphamvu, kukhala ndi nthumanzi, mtima wosweka)? ***Mwachitsanzo****: Kusafuna kukhala kapena kucheza ndi anzanu* | 0 | 1 | 2 | 3 |
| 3. ***M’masabata awiri apitawa***, ndikangati mwakhala mukuvutika kupeza tulo (kulephera kugona kapena kukhala m’maso nthawi yogona) kapena kugona mopitilira/moposa nthawi zonse? | 0 | 1 | 2 | 3 |
| 4. ***M’masabata awiri apitawa***, ndikangati mwakhala mukumva kutopa, kukhala ndi ulesi, kufooka kapena kupelewera/kuchepa mphamvu? ***Mwachitsazo****: Kulephera kupanga ntchito za tsiku ndi tsiku* | 0 | 1 | 2 | 3 |
| 5. ***M’masabata awiri apitawa***, ndikangati mwakhala mukusowa chilakolako cha chakudya kapena kudya kwambiri/mowonjeza? | 0 | 1 | 2 | 3 |
| 6. ***M’masabata awiri apitawa***, ndikangati munaziona ngati opanda pake (osazikhulupilira kapena wolephera kapena kuziona *osafunikila/onyozeka)* komanso kuti mwanyozetsa banja lanu? | 0 | 1 | 2 | 3 |
| 7. ***M’masabata awiri apitawa***, ndikangati munavutika Kulephera kukhazikika pazochita, ***mwachitsanzo***: kulephera kuwerenga kapena kulephera kukhazikika kumvera wailesi kapenanso kuwonera zowonera wonera, kucheza ndi anzanu, kulephera kuchita nawo zokambirana mumsonkhano? | 0 | 1 | 2 | 3 |
| 8. ***M’masabata awiri apitawa***, ndikangati mwakhala mukuyenda kapena kuyankhula pang’onopang’ono kwambiri mpakana anthu ena nkudabwa, kusakhazikika nkumangoyenda yenda moposera muyeso? | 0 | 1 | 2 | 3 |
| 9. ***M’masabata awiri apitawa***, ndikangati mwakhala ndi maganizo akuti kuli bwino kungofa kapena maganizo ofuna kuzivulaza nokha mwanjira ina iliyonse? | 0 | 1 | 2 | 3 |
